# Phylogenomic Analyses of the Genus *Pseudomonas* Lead to the Rearrangement of Several Species and the Definition of New Genera

**DOI:** 10.3390/biology10080782

**Published:** 2021-08-16

**Authors:** Zaki Saati-Santamaría, Ezequiel Peral-Aranega, Encarna Velázquez, Raúl Rivas, Paula García-Fraile

**Affiliations:** 1 Microbiology and Genetics Department, University of Salamanca, 37007 Salamanca, Spain; epa@usal.es (E.P.-A.); evp@usal.es (E.V.); raulrg@usal.es (R.R.); paulagarciafraile@usal.es (P.G.-F.); 2 Institute for Agribiotechnology Research (CIALE), 37185 Salamanca, Spain; 3 Associated Research Unit of Plant-Microorganism Interaction, University of Salamanca-IRNASA-CSIC, 37008 Salamanca, Spain

**Keywords:** *Pseudomonas*, phylogeny, genomics, bacterial taxonomy, comparative genomics, average nucleotide identity, dDDH, *Neopseudomonas*, *Parapseudomonas*, *Denitrificimonas*

## Abstract

**Simple Summary:**

*Pseudomonas* represents a very important bacterial genus that inhabits many environments and plays either prejudicial or beneficial roles for higher hosts. However, there are many *Pseudomonas* species which are too divergent to the rest of the genus. This may interfere in the correct development of biological and ecological studies in which *Pseudomonas* are involved. Thus, we aimed to study the correct taxonomic placement of *Pseudomonas* species. Based on the study of their genomes and some evolutionary-based methodologies, we suggest the description of three new genera (*Denitrificimonas*, *Parapseudomonas* and *Neopseudomonas*) and many reclassifications of species previously included in *Pseudomonas*.

**Abstract:**

*Pseudomonas* is a large and diverse genus broadly distributed in nature. Its species play relevant roles in the biology of earth and living beings. Because of its ubiquity, the number of new species is continuously increasing although its taxonomic organization remains quite difficult to unravel. Nowadays the use of genomics is routinely employed for the analysis of bacterial systematics. In this work, we aimed to investigate the classification of species of the genus *Pseudomonas* on the basis of the analyses of the type strains whose genomes are currently available. Based on these analyses, we propose the creation of three new genera (*Denitrificimonas* gen nov. comb. nov., *Neopseudomonas* gen nov. comb. nov. and *Parapseudomonas* gen nov. comb. nov) to encompass several species currently included within the genus *Pseudomonas* and the reclassification of several species of this genus in already described taxa.

## 1. Introduction

*Pseudomonas* is one of the most diverse and adaptable prokaryotic genera and their metabolic versatility allows their members to survive in many different environments [1,2]. Members of the genus *Pseudomonas* have been identified in human and animal related sources, plants, soil, water environments, psychrophilic environments, and other environmental niches and hosts [3,4,5]. Also, some species of this genus are known to play relevant roles in their hosts, such as *P. aeruginosa*, which causes human lung infections [6], species belonging to the *P. fluorescens* lineage, which are able to promote plants growth [7,8], or even some diverse species suggested to interfere with insects’ biology [9,10], amongst many other cases. The multiplicity of environments where *Pseudomonas* grow and diversify has led to the broad evolution of its members, making it one of the most diverse bacterial genera. *Pseudomonas’* diversity, together with the fact that these bacteria are tremendously versatile and easy to grow under laboratory conditions, has led to the continuous discovery of new species of this genus [4]. However, the large number of species belonging to *Pseudomonas* and related taxa has made their taxonomic classification challenging, involving constant reshaping and reclassifications [11,12].

Moreover, the correct assignment of bacterial species is extremely important for making correct assumptions when carrying out microbiological studies, especially those with ecological relevance [13]. Assigning bacterial functions to wrongly classified taxa could cause future research to be based on false statements [14]; thus, the correct taxonomic allocation of members of this widely spread genus is highly desirable.

During the last two decades, the systematic classification of *Pseudomonas* has been based primarily on 16S rRNA gene sequence comparisons complemented by the construction of phylogenetic trees based on *rpoB*, *rpoD,* and *gyrB* gene sequences [11,15]. Recently, the use of genomic tools has facilitated taxonomic analysis of the genus *Pseudomonas* and the description of many novel *Pseudomonas* species, i.e., [12,16,17]. In these types of studies, the average nucleotide identity (ANI) values and digital DNA-DNA hybridization (dDDH) have both been employed for validating and confirming the taxonomic relatedness among similar and different species, which is always based on the thresholds suggested by several authors [18,19].

Currently, the use of genomics has become so commonplace that genome sequences are usually required for describing and validating novel taxa [19]. As a result, the use of genomics in taxonomy has become known as ‘phylogenomics’ and refers to the computation of phylogenetic trees based on genome-scale approaches. In addition to the overall genome related index (OGRI), which mainly comprises ANI and dDDH indices, several tools and algorithms have recently been developed for the purpose of carrying out phylogenomics [19,20], and have been used to organize and classify a diverse number of taxa through genome-based strategies [21,22,23,24]. In this sense, ANIb values over 95–96% and dDDH values over 70% indicate that a certain pair of bacterial strains represents the same species [19,20]. Also, some authors suggested ANIb < 75–76% for genera delimitation [25,26]. Despite this, there are no well-defined thresholds for this purpose. In addition, average amino acid identities (AAI) are becoming to be calculated in taxonomy for novel genera descriptions; as an example, Ma et al. [27] suggested AAI > 86% as the threshold for genera delimitation in the family *Enterobacteriaceae*. However, there is no consensus on AAI values for the delimitation of species or upper taxonomic levels. Thus, phylogenetics are crucial in these cases, since these help to decipher the evolutionary divergences among different clades.

In addition, Hesse et al. [28] provided the genome sequence of many type strains of the *Pseudomonas* genus and used them to conduct evolutionary studies. In fact, last year, Lalucat et al. [12] used many of these genomes for constructing the phylogeny of *Pseudomonas*, organizing clades into the following diverse groups: *P. anguilliseptica*, *P. straminea*, *P. putida*, *P. syringae*, *P. lutea*, *P. asplenii*, *P. fluorescens*, *P. pertucinogena*, *P. aeruginosa*, *P. resinovorans*, *P. linyingensis*, *P. oleovorans*, *P. stutzeri,* and *P. oryzihabitans*. Also, in a recent study including 494 *Pseudomonas* genomes, some non-type strains for which genomes have been published are suggested to be mis-classified and that should represent different species of *Pseudomonas* or even different genera [29]. However, although many species are distantly related to the core of the genus, there are no published reports suggesting their reclassification in other genera.

Thus, the present study applies phylogenomic approaches to revise the taxonomic organization of the genus *Pseudomonas* through the analysis of public genomes from type strains of species currently included into the genus *Pseudomonas* and other genera of the family *Pseudomonadaceae*. Furthermore, we analyzed the 16S rRNA and housekeeping genes sequences to confirm these rearrangements. The obtained results are in agreement with those of the genome-based phylogeny and OGRI analysis. Altogether, our analyses support the reclassification of several species into new taxa.

Based on the results of this study, we propose the creation of three novel genera to encompass several species currently included into the genus *Pseudomonas* and the reclassification of some *Pseudomonas* species into the genera *Chryseomonas*, *Stenotrophomonas,* and *Xanthomonas*.

## 2. Materials and Methods

### 2.1. Genome Sequence Data and Annotation

The List of Prokaryotic Names with Standing in Nomenclature (LPSN; https://lpsn.dsmz.de/, accessed on 31 August 2020), was used to search for validated type strains of the family *Pseudomonadaceae*, and, when available, their genomic sequences were downloaded from the NCBI database (https://www.ncbi.nlm.nih.gov/, accessed on 31 August 2020). In addition, and owing to their high similarity with some *Pseudomonas* strains, genomes of the genera *Stenotrophomonas* and *Xanthomonas* were also downloaded for taxonomic purposes. Similarly, the genomes of the type strains of other genera were downloaded for further use as phylogenetic tree outgroups.

All genome sequences were uploaded in batch mode to RAST (v2.0) [30] and annotated using the default settings.

Basic statistics for each genomic sequence were obtained using QUAST (v5.0.2) [31] and the graphics were performed in R using the ggplot2 package [32].

### 2.2. Phylogenetics and Phylogenomics

Trees based on the 16S rRNA gene and MLSA based on different housekeeping genes were created using MEGA (v7) [33] as previously described [16,34]. The 16S rRNA and housekeeping genes sequences from all the analyzed type strains were retrieved from RAST annotations when available, or from the NCBI database when the complete sequence of a gene was unavailable in the genome.

In order to assess the similarity of the nucleotide sequences of the 16S rRNA genes of *Pseudomonas* type strains, their sequences were uploaded to EzBioCloud [35], and percentage of similarity data for each strain was recorded.

Phylogenomic trees were constructed using the UBCG (v3) tool (default settings) [36] which created alignments (with MAFFT) and trees based on 92 housekeeping genes. Then, these trees were visualized and edited in The Interactive Tree Of Life (iTOL) tool (v5) [37].

### 2.3. OGRI Analyses

Genome sequence distances were measured by calculating OGRI as previously described [38]. Briefly, the PYANI software (v0.2.10) [39] was employed to obtain blast-based average nucleotide identities (ANIb). A heatmap representing ANIb values was produced with heatmap.2 function from gplots package in R [40]. dDDH values were obtained using the Genome-to-Genome Distance Calculator (GGDC v2.1) tool [41,42] (http://ggdc.dsmz.de/ggdc.php#, accessed on 31 May 2021). Average amino acid identities (AAI) were calculated with EzAAI tool (v1.1) [43] with default settings, which uses MMSeqs2 for protein comparisons, a minimum query coverage of 50% and a minimum identity of 40% for AAI calculations.

### 2.4. Comparative Genomics and a Genome Wide Association Study (GWAS)

For the purpose of carrying out comparative analyses, genomes were annotated using prokka (v1.14.6) [44] and their annotations in General Feature Format (GFF) were used to perform pan-genome calculations and comparisons. To do this, we ran PPanGGOLiN (v1.1.96) using the default settings, except for the MMSeq2 identity threshold employed for clustering, which was set at 0.7 [45]. Then, PPanGGOLiN scripts were used to create a protein presence/absence matrix table in the csv format. Pan-genome plots were created with the roary_plots.py script (https://sanger-pathogens.github.io/Roary/, accessed on 1 February 2021).

GWAS analyses between proteins of different sets of genomes belonging to different phylogenetic clades were performed using Scoary (v1.6.16) [46]; the input consisted in the gene presence/absence matrix, traits tables (0–1 binary code) that identified genomes with clades. The output tables were examined to identify proteins exclusively present or absent in certain phylogenetic clades. The COG family of these proteins were assigned using eggNOG-mapper (v5) [47].

## 3. Results and Discussion

### 3.1. Phylogenomics in Pseudomonadaceae

To obtain a more global picture of the taxonomic organization of the genus *Pseudomonas*, genomes of type strains from the family *Pseudomonadaceae* were downloaded, whose genomic data are summarized in Table S1.

**Figure 1:**
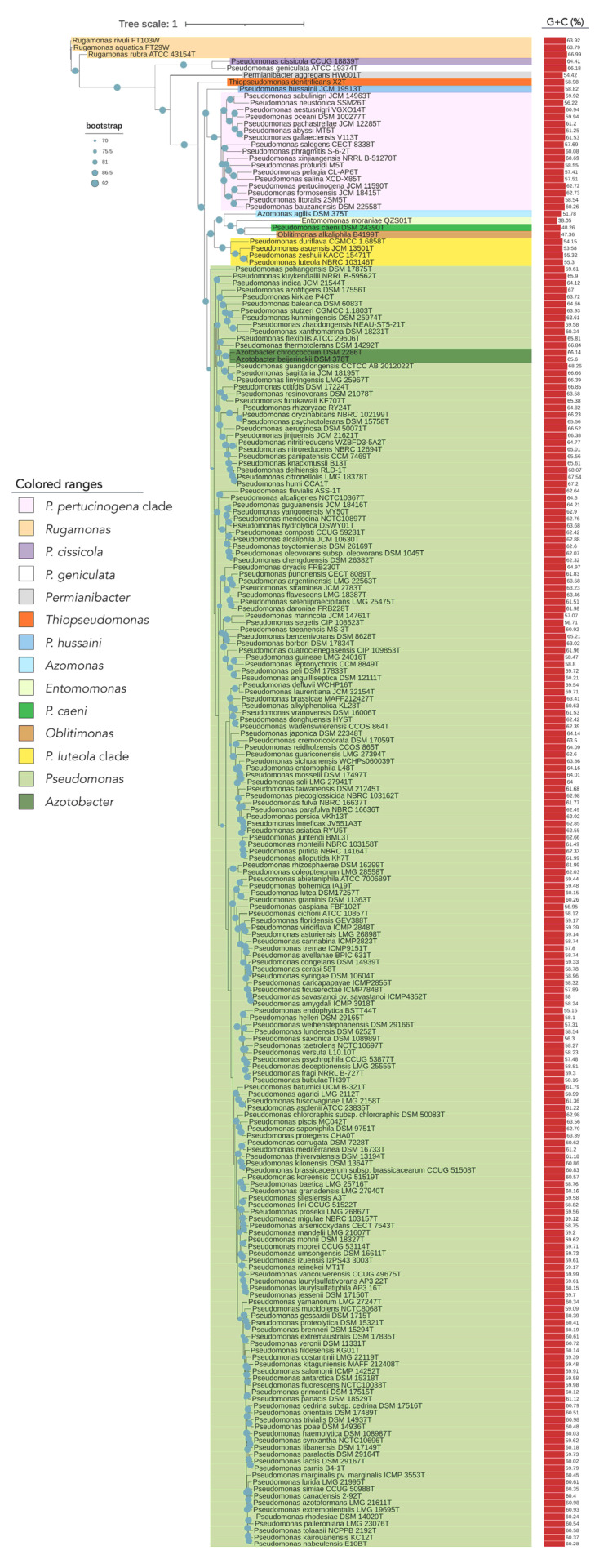
Phylogenomic tree of the *Pseudomonadaceae* type strains based on 92 concatenated housekeeping genes. The tree was built with UBCG, which uses MAFFT to create the multi-gene alignment and FastTree for computing the tree. Red bars represent GC (mol%) content for each genome. Blue circles represent bootstrap values, which indicate the number of individual phylogenetic trees of each of the 92 genes that support each branch (Gene Support Index). Bootstrap value of 92 means that the branch is supported by all UBCG phylogenies.

**Figure 2:**
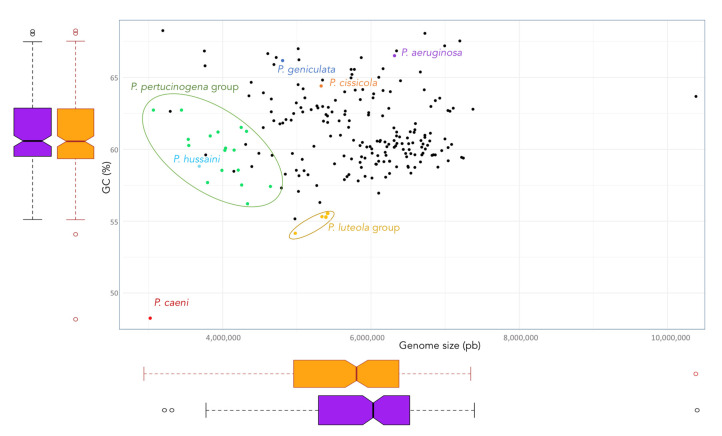
Size and G + C (mol%) content of the genomes of the *Pseudomoans* type strains. Boxplots represents these genomic features of the genus (orange) and those that remain when exclude the genomes of the type strains of *P. hussaini*, *P. caeni*, *P. geniculata*, *P. cissicola*, and those of the *P. luteola* and *P. pertucinogena* clades (purple).

The phylogenomic tree built on the basis on 92 housekeeping genes from the genomes of all type strains of the genus *Pseudomonas* and related genera is shown in Figure 1. This tree distinguishes some *Pseudomonas* lineages and clades that are differentiated from the big clade that clusters most *Pseudomonas* type strains, including that of *P. aeruginosa*, the type species of the genus (Figure 1). Some of these more divergent type strains have a different genome size and G + C mol% content (Figure 2). OGRIs values shows that they have lower ANIb values with those type strains included in the main *Pseudomonas* clade than some thresholds that have been suggested for genera delimitation (75–76%) [25,26] (Table S2). On the other hand, some pairs of type species display higher ANIb or dDDH values than those suggested for species differentiation, which are 95–96% in the case of ANIb and 70% in the case of dDDH.

**Figure 3:**
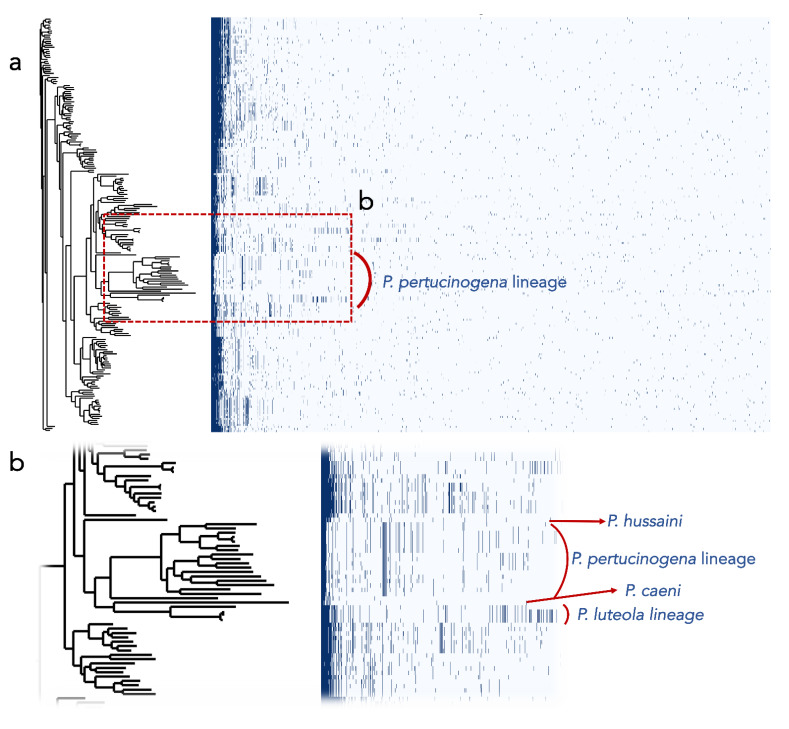
(**a**) Presence/absence profiles of the protein clusters of the genomes of the *Pseudomonas* type strains. The phylogenetic tree was built with UBCG. (**b**) Screen enlargement showing the protein profiles of the main lineages that are divergent from the other genomes of *Pseudomonas*.

Additionally, we found that the encoded proteins in the genomes of the most divergent clades or lineages differ substantially from those of the remaining *Pseudomonas* (Figure 3). Out of the protein clusters generated in the pangenomic analysis, the *P. pertucinogena* clade had 316 protein families which were unique to this lineage, as they were absent in any other *Pseudomonas* species (Figure 3 and Figure S1). Likewise, *P. caeni* DSM 24390^T^, *P. hussainii* JMC 19513^T^, and the *P. luteola* clade had 1462, 1784, and 708 unique proteins, respectively. To know what metabolic categories differentiate these divergent clades from the remaining *Pseudomonas,* these proteins were assigned to Clusters of Orthologous Groups of proteins (COGs). The majority of them were either hypothetical proteins or proteins included within the categories “Cell wall/membrane/envelope biogenesis”, “Amino acid transport and metabolism”, “Signal transduction mechanisms” or “Transcription” (Figure S1). On the contrary, as depicted in Figure 3, several protein families abundant in *Pseudomonas* belonging to the main clade were absent in the *P. pertucinogena* and *P. luteola* clades.

### 3.2. Taxonomic Status of Phylogenetically Distant Lineages and Clades

Considering all above-mentioned analyses, a case-by-case summary of the situation of those phylogenetically distant lineages and clades to the main *Pseudomonas* group is presented below. Based on that, we resolve a more appropriate taxonomic classification for each of them.

#### 3.2.1. *Pseudomonas geniculata*

*P. geniculata* ATCC 19374^T^ forms a lineage in the *Pseudomonadaceae* phylogenomic tree highly divergent from other *Pseudomonas* strains and those of the related genera. Thus, we compared its 16S rRNA gene sequence with those of the type strains held in the NCBI database, finding that it is more closely related to *Stenotrophomonas* species than to *Pseudomonas* ones. This was already pointed out previously: Anzai et al. [11] and Ramos et al. [48], who placed it within the genus *Stenotrophomonas*, although this reclassification has never been validly published.

**Figure 4:**
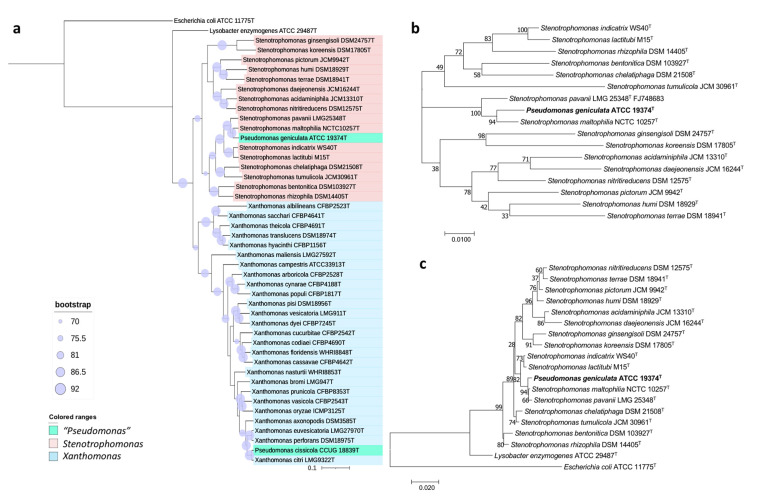
Phylogenetic trees built to study the taxonomic placement of *P. geniculata* ATCC 19374^T^ based on (**a**) 92 housekeeping genes, (**b**) 16S rRNA and gyrB concatenated genes, (**c**) the 16S rRNA gene. Scale bars = 1 nucleotide (nt) substitution per 100 nt. Blue circles represent bootstrap values, which indicate the number of individual phylogenetic trees of each of the 92 genes that support each branch (Gene Support Index). Bootstrap value of 92 means that the branch is supported by all UBCG phylogenies.

Hence, we performed diverse approaches to settle the taxonomic placement of this type species. The 16S rRNA-based phylogeny placed *P. geniculata* ATCC 19374^T^ within the genus *Stenotrophomonas*, together with *S. maltophilia* NCTC 10257^T^ and *S. pavanii* LMG 25348^T^ (Figure 4c), sharing with them a 99.6 and 99.8% sequence similarity, respectively. These results are in concordance with the phylogeny based on concatenated 16S rRNA and *gyrB* genes (Figure 4b) and the UBCG tree (Figure 4a). The ANIb and dDDH values shared among *P. geniculata* ATCC 19374^T^ and *Stenotrophomonas* strains were below the threshold values used for species delimitation [18,19] (Tables S3 and S4). Concretely, values of ANIb and dDDH between *P. geniculata* ATCC 19374^T^ and *S. maltophilia* NCTC 10257^T^ are 92.37% and 48.5%, respectively, and those between *P. geniculata* ATCC 19374^T^ and *S. pavanii* LMG 25348^T^ are 42.3% and 90.75%. Altogether, the results of OGRI, phylogenetic, and phylogenomic analyses allow us to propose the reclassification of *P. geniculata* as *Stenotrophomonas geniculata* comb. nov.

#### 3.2.2. *Pseudomonas cissicola*

*P. cissicola* CCUG 18839^T^ was also clearly differentiated from the clade including most of the *Pseudomonas* species (Figure 1). The 16S rRNA BLASTn-based gene identification placed this strain within the genus *Xanthomonas*.

Hu et al. [49] already reported many phenotypic and chemotaxonomic similarities between *P. cissicola* CCUG 18839^T^ and *Xanthomonas* species. This was also already pointed out by Anzai et al. [11]. Later, Parkinson et al. [50] performed phylogenetic approaches which located *P. cissicola* in the *Xanthomonas citri* subsp. *citri* clade.

**Figure 5:**
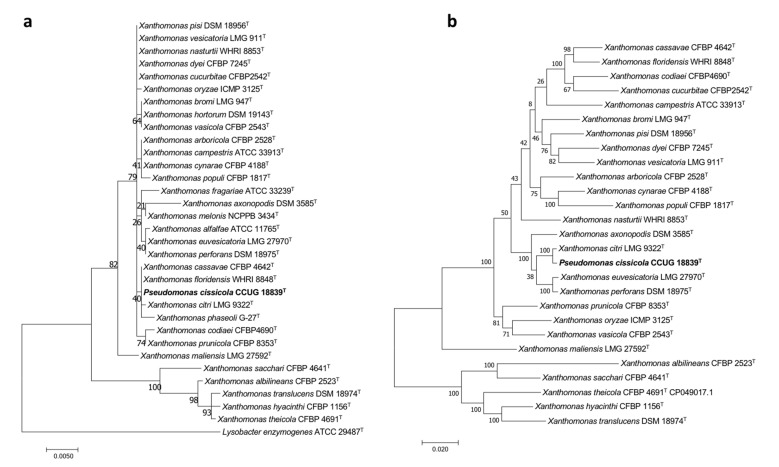
Phylogenetic trees built to study the taxonomic placement of *P. cissicola* CCUG 18839^T^ based on (**a**) the 16S rRNA gene (**b**) 16S rRNA, *gyrB*, *rpoD*, *dnaK* and *fyuA* concatenated genes. Scale bars = 1 nucleotide (nt) substitution per 100 nt.

The phylogenetic trees of this work were built UBCG (Figure 4a) and those built with the sequences of both the 16S rRNA gene and the concatenated sequences of the housekeeping genes *gyrB*, *rpoD*, *dnaK*, and *fyuA* (Figure 5) clustered *P. cissicola* CCUG 18839^T^ together with *Xanthomonas citri* LMG9322^T^, with *X. campestris*, *X*, *axonopodis, X. euvesicatoria,* and *X. perforans* also closely related. *P. cissicola* CCUG 18839^T^ and *Xanthomonas citri* LMG9322^T^ share ANIb and dDDH values of 98.3% and 86.7%, respectively (Tables S4 and S5), which are above the thresholds for species differentiation [18,19]. These values are lower than 94.0% and 55.8%, respectively between *P. cissicola* CCUG 18839^T^ and any of the remaining *Xanthomonas* species (Tables S4 and S5). Therefore, the high similarity between the type strains of *P. cissicola* and *Xanthomonas citri* supports the reclassification of the species *P. cissicola* into the genus *Xanthomonas* as a later synonym of *Xanthomonas citri*.

#### 3.2.3. *Pseudomonas caeni*

**Figure 6:**
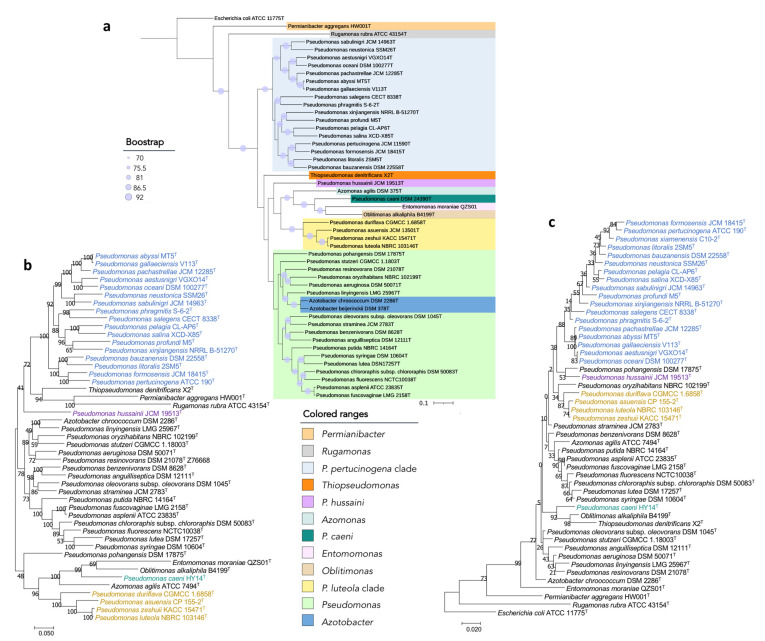
Phylogenetic trees built to study the taxonomic placement of divergent *Pseudomonas* type strains based on (**a**) 92 housekeeping genes, (**b**) 16S rRNA, *gyrB*, *rpoB* and *rpoD* concatenated genes, (**c**) the 16S rRNA gene. Scale bars = 1 nucleotide (nt) substitution per 100 nt. Blue circles represent bootstrap values, which indicate the number of individual phylogenetic trees of each of the 92 genes that support each branch (Gene Support Index). Bootstrap value of 92 means that the branch is supported by all UBCG phylogenies.

**Figure 7:**
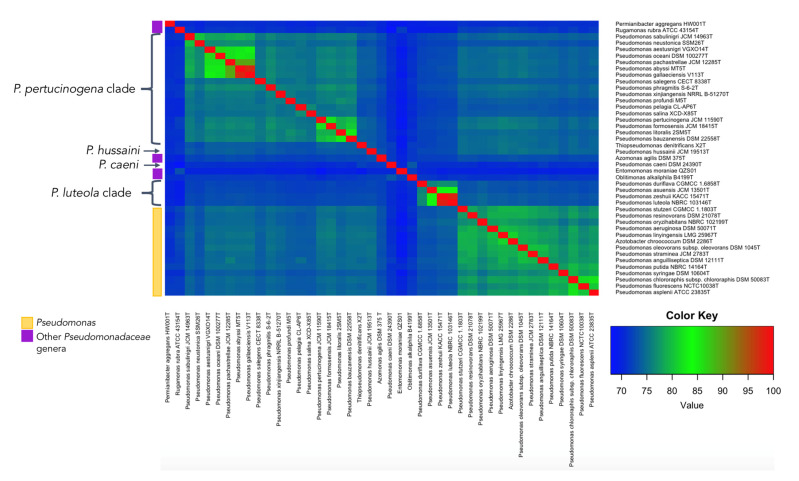
Heatmap of ANIb values calculated for the genomes of the type strains suggested as novel genera and the genomes of other representative *Pseudomonadaeae* type strains.

*P. caeni* DSM 24390^T^ was located on a separate branch from the remaining *Pseudomonas* species in all phylogenetic trees constructed in this study (Figure 1 and Figure 6). The analysis of the ANIb values (lower than 73.4% in all cases) and AAI values (lower than 70.9%) also showed large differences between the genomes of *P. caeni* DSM 24390^T^ and those of the remaining members of the family *Pseudomonadaceae* (Tables S2 and S6 and Figure 7). Indeed, genome size and G + C mol% content of *P. caeni* DSM 24390^T^ are extremely divergent from other type strains of the genus *Pseudomonas* (Figure 2), as it has been recently reported [28]. Therefore, given the results obtained in the present work *P. caeni* should be transferred to a new genus for which we propose the name *Denitrificimonas* gen. nov. because of its denitrifying potential, being *Denitrificimonas caeni* gen. nov. comb. nov. its type species.

#### 3.2.4. *P. hussainii*

*P. hussainii* JMC 19513^T^ is well differentiated from its closest related taxa in all the constructed phylogenetic and phylogenomic trees of the family *Pseudomonadaceae* (Figure 1 and Figure 6). The ANIb and AAI values between *P. hussaini* JMC 19513^T^ and other *Pseudomonas* species are below 75.3% and 70%, respectively (Tables S2 and S6 and Figure 7). Therefore, we propose the reclassification of the species *P. hussainii* in the novel genus *Parapseudomonas* gen. nov. as *Parapseudomonas hussainii* gen. nov. comb. nov.

#### 3.2.5. *Pseudomonas luteola* Clade

This clade encompasses the species *P. luteola*, *P. asuensis*, *P. zeshuii,* and *P. duriflava*. The phylogenetic analyses based on the 16S rRNA genes, *gyrB*, *rpoB,* and *rpoD* housekeeping genes and 92 housekeeping genes selected using the UBCG tool (Figure 1 and Figure 6) clearly separate the type species of this clade from all the remaining genera included in the family *Pseudomonadaceae*. The ANIb and AAI values between each one of the type strains of this clade and those of the other *Pseudomonadaceae* type strains were lower than 75% and 73% (Tables S2 and S6 and Figure 7) and the similarity values of 16S rRNA gene sequences were equal to or lower than 97%.

At the same time, the type species of this group (Figure 1 and Figure 6) showed among them ANIb values ranging from 76% to 95%, except in the case of *P. zeshuii* KACC 15471^T^ and *P. luteola* NBRC 103146^T^, which showed an ANIb value of 97.9% and a dDDH value of 92.0% (Tables S2 and S3). Therefore, as previously suggested by Lalucat et al. [12], *P. zeshuii* is a later synonym of *P. luteola*.

The species *P. luteola* was transferred to the genus *Chryseomonas* in year 1987 based on its DNA relatedness with *Chryseomonas polytricha* [51] and transferred again to the genus *Pseudomonas* in 1997 based on the 16S rRNA gene sequence homology with this genus [52]. However, all analyses performed in this study showed that the clade of *P. luteola* corresponds to a different genus from *Pseudomonas*. Thus, the initial reclassification of the species into the genus *Chryseomonas,* as *C. luteola,* should be maintained. Based on this, we propose to transfer to the genus *Chryseomonas* the species *P. asuensis* and *P. duriflava* as *Chryseomonas asuensis* comb. nov. and *Chryseomonas duriflava* comb. nov.

#### 3.2.6. *Pseudomonas pertucinogena* Clade

The clade of *P. pertucinogena* currently includes 18 *Pseudomonas* species (see Figure 1 and Figure 6 and Table S2). The phylogenetic trees based on genome sequences, 16S rRNA gene sequences and the sequences of concatenated housekeeping genes *gyrB*, *rpoB*, and *rpoD* located all species of the *P. pertucinogena* clade in a divergent taxonomic group within the family *Pseudomonadaceae* (Figure 6). The 16S rRNA gene similarities, ANIb and AAI values between any of the type strains of the clade and those of the remaining type strains of the family are lower than 95%, 75%, and 67%, respectively (Tables S2 and S6 and Figure 7), which suggests that the clade is substantially divergent from the *Pseudomonas* genus. This was also reported by Lalucat et al. [12] who suggested that the cluster of *P. pertucinogena* should be considered a different genus of the *Pseudomonadaceae* family based on phylogenetic approaches. All species from this group showed ANIb values among them lower than 95%, except in the case of *P. abyssi* and *P. gallaeciensis* which showed an ANIb value of 97,6 and a dDDH value of 80.1% (Table S3). These results agree with those of Lalucat et al. [12], who suggested the synonymy of the names *P. abyssi* and *P. gallaeciensis*. Since the name *P. abyssi* has priority, *P. gallaeciensis* is a later synonym of *P. abyssi*. Considering the results obtained in this work, we propose the creation of the novel genus *Neopseudomonas* gen. nov. for the remaining 17 species of the *P. pertucinogena* clade, with 17 new combinations.

## 4. Conclusions

This work highlights the relevance of the use of genomics in prokaryotic taxonomy. The phylogenetic and phylogenomic analyses performed in this study provide an overview of the genus *Pseudomonas* showing misclassified species or lineages. Phylogenetics and phylogenomics, together with OGRI values, outperform the resolution of 16S rRNA and housekeeping genes analyses to clarify the taxonomic organization of a broad genus such as *Pseudomonas*. These analyses allowed the reclassification of some previous *Pseudomonas* species into newly defined genera and into other already defined ones.

## 5. Description of New Taxa

### 5.1. Description of Stenotrophomonas geniculata comb. nov.

ge.ni.cu.la’ta. (L. fem. adj. *geniculata*, bent at a sharp angle).

Basonym: *Pseudomonas geniculata* (Wright 1895) Chester 1901 (Approved Lists 1980).

The description is as given for *Pseudomonas geniculata* [53] with the following modification. The genomic G + C content of the type strain is 66.2% and its genomic size is approximately 4.81 Mbp. The type strain is ATCC 19374 = JCM 13324 = LMG 2195 = NCIB 9428 = NCIMB 9428.

### 5.2. Description of Denitrificimonas gen. nov.

De.ni.tri.fi.ci.mo’nas. (N.L. v. *denitrifico*, to denitrify; L. fem. n. *monas*, a unit, monad; N.L. fem. n. *Denitificimonas*, a denitrifying monad).

The description is as given for *Denitificimonas caeni*, which is the type species. The genus has been separated from *Pseudomonas* based on the physiology and phylogenetic analyses of genome and 16S rRNA gene sequences.

### 5.3. Description of Denitrificimonas caeni comb. nov.

cae’ni. (L. gen. neut. n. *caeni*, of sludge).

Basonym: *Pseudomonas caeni* Xiao et al., 2009.

The description is as given for *Pseudomonas caeni* [54] with the following modification. The genomic G + C content of the type strain is 48.3% and its genomic size is approximately 3.02 Mbp. The type strain is HY-14 = DSM 24390 = KCTC 22292 = CCTCC AB208156.

### 5.4. Description of Parapseudomonas gen nov.

Pa.ra.pseu.do.mo’nas. (Gr. pref. *para-*, besides, alongside of; N.L. fem. n. *Pseudomonas* a bacterial genus; N.L. fem. n. *Parapseudomonas*, a genus adjacent to *Pseudomonas*).

The description is as given for *Parapseudomonas hussainii*, which is the type species. The genus has been separated from *Pseudomonas* based on phylogenetic analyses of genome and 16S rRNA gene sequences.

### 5.5. Description of Parapseudomonas hussainii comb. nov.

hus.sai’ni.i. (N.L. gen. masc. n. *hussainii*, named after S. A. Hussain, an Indian ornithologist and avian gut biologist).

Basonym: *Pseudomonas hussainii* Hameed et al., 2014.

The description is as given for *Pseudomonas hussainii* [55] with the following modification. The genomic G + C is 58.8% and its genomic size is approximately 3.68 Mbp. The type strain is CC-AMH-11 = JCM 19513 = BCRC 80696.

### 5.6. Description of Chryseomonas asuensis comb. nov.

a.su.en’sis. (N.L. fem. adj. *asuensis*, an adjective arbitrarily derived from Arizona State University).

Basonym: *Pseudomonas asuensis* Reddy and Garcia-Pichel 2015.

The description is as given for *Chryseomonas asuensis* [56] with the following modification. The genomic G + C is 53.6% and its genomic size is approximately 5.36 Mbp. The type strain is CP155-2 = DSM 17866 = ATCC BAA-1264 = JCM13501 = KCTC 32484.

### 5.7. Description of Chryseomonas duriflava comb. nov.

du.ri.fla’va. (L. masc. adj. *durus*, hard; L. masc. adj. *flavus*, yellow; N.L. fem. adj. *duriflava*, hard yellow).

Basonym: *Pseudomonas duriflava* Liu et al., 2008.

The description is as given for *Pseudomonas duriflava* [57] with the following modification. The genomic G + C is 54.2% and its genomic size is about 4.98 Mbp. The type strain is HR2 = CGMCC 1.6858 = DSM 21419 = KCTC 22129.

### 5.8. Description of Neopseudomonas gen. nov.

Ne.o.pseu.do.mo’nas. (Gr. masc. adj. *neos*, new; N.L. fem. n. *Pseudomonas*, a bacterial genus; N.L. fem. n. *Neopseudomonas*, a new group of *Pseudomonas*).

Gram negative, motile, and non-spore-forming bacteria. Cells are rod-shaped. Aerobic. Catalase and oxidase positive. The major fatty acids are those from Summed feature 8 (C_18:1_ *ω*6*c* and/or C_18:1_ *ω*7*c*) and the main respiratory quinone is Q9. The G + C content as calculated from genome sequences is approximately 60% and its genome size, 4.0 Mbp. The type species is *Neopseudomonas pertucinogena* comb. nov.

### 5.9. Description of Neopseudomonas abyssi comb. nov.

a.bys′si. (N.L gen. n. *abyssi*, of an abyss).

Basonym: *Pseudomonas abyssi* Wei et al., 2018.

The description is as given for *Pseudomonas abyssi* [58] with the following modification. The genomic G + C is 61.3% and its genomic size is approximately 4.32 Mbp. The type strain is MT5 = KCTC 62295 = MCCC 1K03351.

### 5.10. Description of Neopseudomonas aestusnigri comb. nov.

aes.tus.ni’gri. (L. masc. adj. *aestus*, tide; L. masc. adj. *niger*, black; N.L. gen. n. *aestusnigri*, of black tide).

Basonym: *Pseudomonas aestusnigri* Sánchez et al., 2014.

The description is as given for *Pseudomonas aestusnigri* [59] with the following modification. The genomic G + C is 60.4% and its genomic size is about 3.83 Mbp. The type strain is VGXO14 = CCUG 64165 = CECT 8317.

### 5.11. Description of Neopseudomonas bauzanensis comb. nov.

bau.za.nen’sis. (N.L. fem. adj. *bauzanensis*, of or belonging to Bauzanum medieval Latin name of Bozen/Bolzano, a city in South Tyrol, Italy, where the species was first isolated).

Basonym: *Pseudomonas bauzanensis* Zhang et al., 2011.

The description is as given for *Pseudomonas bauzanensis* [60] with the following modification. The genomic G + C is 60.3% and its genomic size is approximately 3.54 Mbp, The type strain is BZ93 = CGMCC 1.9095 = DSM 22558 = LMG 26048.

### 5.12. Description of Neopseudomonas formosensis comb. nov.

for.mo.sen’sis. (N.L. fem. adj. *formosensis*, of or pertaining to Formosa (Taiwan), the beautiful island).

Basonym: *Pseudomonas formosensis* Lin et al., 2013.

The description is as given for *Pseudomonas formosensis* [61] with the following modification. The genomic G + C is 62.7% and its genomic size is approximately 3.44 Mbp. The type strain is CC-CY503 = BCRC 80437 = JCM 18415.

### 5.13. Description of Neopseudomonas litoralis comb. nov.

li.to.ra’lis. (L. fem. adj. *litoralis*, of or belonging to the seashore).

Basonym: *Pseudomonas litoralis* Pascual et al., 2012.

The description is as given for *Pseudomonas litoralis* [62] with the following modification. The genomic G + C is 58.5% and its genomic size is approximately 3.99 Mbp The type strain is 2SM5 = CECT 7670 = DSM 26168 = KCTC 23093.

### 5.14. Description of Neopseudomonas neustonica comb. nov.

neus.to’ni.ca. (N.L. fem. adj. *neustonica*, pertaining to and living in the neuston).

Basonym: *Pseudomonas neustonica* Jang et al., 2020.

The description is as given for *Pseudomonas neustonica* [63]. The genomic G + C is 56.2% and its genomic size is approximately 4.33 Mbp. The type strain is SSM26 = KCCM 43193 = JCM 31284.

### 5.15. Description of Neopseudomonas oceani comb. nov.

o.ce.a’ni. (L. gen. masc. n. *oceani*, of the ocean).

Basonym: *Pseudomonas oceani* Wang and Sun 2016.

The description is as given for *Pseudomonas oceani* [64] with the following modification. The genomic G + C is 59.9% and its genomic size is approximately 4.16 Mbp. The type strain is KX 20 = CGMCC 1.15195 = DSM 100277.

### 5.16. Description of Neopseudomonas pachastrellae comb. nov.

pa.chas.trel’lae. (L. gen. fem. n. *pachastrellae*, of *Pachastrella*, the generic name of a sponge).

Basonym: *Pseudomonas pachastrellae* Romanenko et al., 2005.

The description is as given for *Pseudomonas pachastrellae* [65] with the following modification. The genomic G + C is 61.2% and its genomic size is approximately 3.93 Mbp. The type strain is KMM 330 = CCUG 46540 = DSM 17577 = JCM 12285 = NRIC 583.

### 5.17. Description of Neopseudomonas pelagia comb. nov.

pe.la’gi.a. (L. fem. adj. *pelagia*, of the sea).

Basonym: *Pseudomonas pelagia* Hwang et al., 2009.

The description is as given for *Pseudomonas pelagia* [66] with the following modification. The genomic G + C is 57.4% and its genomic size is approximately 4.64 Mbp. The type strain is CL-AP6 = DSM 25163 = JCM 15562 = KCCM 90073.

### 5.18. Description of Neopseudomonas pertucinogena comb. nov.

per.tu.ci.no’ge.na. (N.L. neut. n. *pertucinum*, pertucin, a bacteriocin that inhibits smooth strains of *Bordetella pertussis*; Gr. v. *gennao*, to produce; N.L. fem. adj. *pertucinogena*, producing pertucin).

Basonym: *Pseudomonas pertucinogena* Kawai and Yabuuchi 1975 (Approved Lists 1980).

The description is as given for *Pseudomonas pertucinogena* [67] with the following modification. The genomic G + C is 62.7% and its genomic size is approximately 3.07 Mbp. The type strain is ATCC 190 = CCUG 7832 = CIP 106696 = DSM 18268 = IFO 14163 = JCM 11590 = LMG 1874 = NBRC 14163.

### 5.19. Description of Neopseudomonas phragmitis comb. nov.

phrag.mi’tis. (L. gen. n. *phragmitis* of reed, of the plant genus *Phragmites*).

Basonym: *Pseudomonas phragmitis* Li et al., 2020.

The description is as given for *Pseudomonas phragmitis* [68] with the following modification. The genomic G + C is 60.1% and its genomic size is approximately 4.04 Mbp. The type strain is S-6-2 = CGMCC 1.15798 = KCTC 52539.

### 5.20. Description of Neopseudomonas profundi comb. nov.

pro.fun’di. (L. gen. neut. n. *profundi*, of the depths of the sea, of the deep sea).

Basonym: *Pseudomonas profundi* Sun et al., 2018.

The description is as given for *Pseudomonas profundi* [69] with the following modification. The genomic G + C is 58.6% and its genomic size is approximately 4.21 Mbp. The type strain is M5 = CCTCC AB 2017186 = CICC 24308 = KCTC 62119.

### 5.21. Description of Neopseudomonas sabulinigri comb. nov.

sa.bu.li.ni’gri. (L. neut. n. *sabulum* sand; L. masc. adj. *niger*, black; N.L. gen. n. *sabulinigri* of black sand).

Basonym: *Pseudomonas sabulinigri* Kim et al., 2009.

The description is as given *Pseudomonas sabulinigri* [70] with the following modification. The genomic G + C is 59.9% and its genomic size is approximately 4.03 Mbp. The type strain is J64 = DSM 23971 = JCM 14963 = KCTC 22137.

### 5.22. Description of Neopseudomonas salegens comb. nov.

sal.e’gens. (L. masc. n. *sal*, salt; L. pres. part. *egens*, being in need; N.L. part. adj. *salegens*, being in need of salt).

Basonym: *Pseudomonas salegens* Amoozegar et al., 2014.

The description is as given for *Pseudomonas salegens* [71] with the following modification. The genomic G + C is 57.7% and its genomic size is approximately 3.80 Mbp. The type strain is GBPy5 =CECT 8338 = IBRC-M 10762.

### 5.23. Description of Neopseudomonas salina comb. nov.

sa.li’na. (N.L. fem. adj. *salina*, salty).

Basonym: *Pseudomonas salina* Zhong et al., 2015.

The description is as given for *Pseudomonas salina* [72] with the following modification. The genomic G + C is 57.5% and its genomic size is approximately 4.26 bp. The type strain is XCD-X85 = CGMCC 1.12482 = JCM 19469.

### 5.24. Description of Neopseudomonas xinjiangensis comb. nov.

xin.jiang.en’sis. (N.L. fem. adj. *xinjiangensis*, pertaining to Xinjiang, in north-west China, where the type strain was isolated).

Basonym: *Pseudomonas xinjiangensis* Liu et al., 2009.

The description is as given for *Pseudomonas xinjiangensis* [73] with the following modification. The genomic G + C is 60.7% and its genomic size is about 3.54 Mbp. The type strain is S3-3 = CCTCC AB 207151 = DSM 23391 = NRRL B-51270.

## Data Availability

Not applicable.

## Supplementary Materials

The following are available online at https://www.mdpi.com/article/10.3390/biology10080782/s1, Figure S1: Heatmap of COG categories of unique proteins of the divergent *Pseudomonas* type strains, Table S1: Genomic features of the type strains used in this study, Table S2: ANIb values shared among *Pseudomonas* type strains that are proposed as novel genera and representative type strains belonging to different *Pseudomonas* and *Pseudomonadaceae* clades, Table S3: ANIb values shared among different *Stenotrophomonas* type strains and *P. geniculata* ATCC 19374^T^. Table S4: Digital DNA-DNA Hybridization (dDDH) values of different type strains that are studied for its taxonomic relocation. Table S5: ANIb values shared among different *Xanthomonas* type strains and *P. cissicola* CCUG 18839^T^. Table S6: AAIb values shared among *Pseudomonas* type strains that are proposed as novel genera and representative type strains belonging to different *Pseudomonas* and *Pseudomonadaceae* clades.
