# Supplementary material for: Phylogenomic Analyses of the Genus Pseudomonas Lead to the Rearrangement of Several Species and the Definition of New Genera
Source: Biology (Basel). 2021 Aug 16;10(8):782. doi: 10.3390/biology10080782 (PMC8389581; doi:10.3390/biology10080782)
Supplement: Supplementary file 1 [file biology-10-00782-s001.zip › biology-1314851/supplementary files/Table S3.pdf]

Table S3: ANIb values shared among different *Stenotrophomonas* type strains and *P. geniculata* ATCC 19374^{T}

	S. rhizophila DSM14405^{T}	*S. pavanii* LMG25348^{T}	*S. nitritireducens* DSM12575^{T}	*S. koreensis* DSM17805^{T}	*P. geniculata* ATCC 19374^{T}	*S. pictorum* JCM9942^{T}	*S. ginsengisoli* DSM24757^{T}	*S. daejeonensis* JCM16244^{T}	*S. bentonitica* DSM103927^{T}	*S. indicatrix* WS40^{T}	*S. acidaminiphila* JCM13310^{T}	*S. humi* DSM18929^{T}	*S. lactitubi* M15^{T}	*S. chelatiphaga* DSM21508^{T}	*S. maltophilia* NCTC10257^{T}	*S. terrae* DSM18941^{T}	*S. tumulicola* JCM30961^{T}
S. rhizophila DSM14405^{T}	100	81.93	81.14	77.92	81.87	80.26	78.06	81.11	85.51	82.01	81.01	79.89	81.83	81.68	81.91	79.86	81.20
S. pavanii LMG25348^{T}	81.85	100	80.83	77.74	90.85	79.65	77.76	80.81	81.90	86.77	80.80	79.26	86.82	82.46	90.99	79.48	82.11
S. nitritireducens DSM12575^{T}	81.09	81.03	100	78.83	80.90	83.87	78.82	86.77	81.50	80.69	89.75	82.62	80.64	80.35	81.14	82.68	79.94
S. koreensis DSM17805^{T}	77.95	77.94	78.86	100	77.89	78.23	84.52	78.78	77.83	77.81	78.82	77.73	77.68	77.55	78.02	78.12	77.23
P. geniculata ATCC 19374^{T}	81.71	90.75	80.79	77.86	100	79.93	77.91	80.69	81.86	86.79	80.60	79.35	86.88	82.28	92.37	79.41	82.10
S. pictorum JCM9942^{T}	80.08	79.78	83.98	78.04	79.84	100	78.18	83.11	80.21	79.68	83.14	82.87	79.49	79.29	79.91	83.44	79.10
S. ginsengisoli DSM24757^{T}	78.18	78.05	78.86	84.52	78.09	78.20	100	78.81	77.90	77.90	78.68	77.71	77.79	77.70	78.04	78.12	77.38
S. daejeonensis JCM16244^{T}	81.10	81.00	86.88	78.86	80.75	83.14	78.78	100	81.09	80.64	86.54	82.65	80.50	80.44	80.80	82.64	79.97
S. bentonitica DSM103927^{T}	85.46	81.93	81.50	77.61	81.85	80.18	77.75	81.03	100	81.77	81.18	79.62	81.87	81.40	81.81	79.68	81.02
S. indicatrix WS40^{T}	81.94	86.77	80.60	77.65	86.86	79.63	77.74	80.62	81.64	100	80.48	79.36	93.45	82.46	86.76	79.54	82.01
S. acidaminiphila JCM13310^{T}	80.77	80.80	89.78	78.68	80.61	83.02	78.55	86.40	81.20	80.45	100	82.35	80.74	80.11	80.81	82.32	79.66
S. humi DSM18929^{T}	79.75	79.40	82.69	77.59	79.39	82.95	77.66	82.45	79.60	79.41	82.41	100	79.25	79.07	79.47	83.46	78.79
S. lactitubi M15^{T}	81.73	86.82	80.43	77.54	86.88	79.48	77.62	80.30	81.82	93.37	80.50	79.20	100	82.50	86.79	79.39	82.05
S. chelatiphaga DSM21508^{T}	81.73	82.52	80.43	77.67	82.36	79.38	77.72	80.36	81.54	82.56	80.29	79.08	82.48	100	82.50	79.19	84.88
S. maltophilia NCTC10257^{T}	81.85	91.01	81.01	77.97	92.50	80.20	77.95	80.90	81.96	86.78	80.93	79.50	86.84	82.56	100	79.71	82.32
S. terrae DSM18941^{T}	79.92	79.59	82.76	77.98	79.55	83.42	77.89	82.51	79.74	79.59	82.40	83.39	79.50	79.20	79.60	100	78.68
S. tumulicola JCM30961^{T}	80.98	82.06	79.97	77.40	82.01	79.07	77.25	79.76	80.96	81.99	79.71	78.65	81.97	84.69	82.16	78.75	100
