# Supplementary material for: Phylogenomic Analyses of the Genus Pseudomonas Lead to the Rearrangement of Several Species and the Definition of New Genera
Source: Biology (Basel). 2021 Aug 16;10(8):782. doi: 10.3390/biology10080782 (PMC8389581; doi:10.3390/biology10080782)
Supplement: Supplementary file 1 [file biology-10-00782-s001.zip › biology-1314851/supplementary files/Table S5.pdf]

Table S5: ANIb values shared among different *Xanthomonas* type strains and *P. cissicola* CCUG 18839^T

	X. oryzae ICMP3125^T	*X. codiaei* CFBP4690^T	*X. maliensis* LMG27592^T	*X. cassavae* CFBP4642^T	*X. bromi* LMG947^T	*X. cucurbitae* CFBP2542^T	*X. dyei* CFBP7245^T	*X. prunicola* CFBP8353^T	*X. hyacinthi* CFBP1156^T	*X. albilineans* CFBP2523^T	*X. citri* LMG9322^T	*X. campestris* ATCC33913^T	*X. cynarae* CFBP4188^T	*X. euvesicatoria* LMG27970^T	*X. floridensis* WHRI8848^T	*X. vasicola* CFBP2543^T	*X. arboricola* CFBP2528^T	*X. translucens* DSM18974^T	*X. sacchari* CFBP4641^T	*X. axonopodis* DSM3585^T	*X. nasturtii* WHRI8853^T	*P. cissicola* CCUG 18839^T	*X. pisi* DSM18956^T	*X. perforans* DSM18975^T	*X. theicola* CFBP4691^T	*X. vesicatoria* LMG911^T	*X. populi* CFBP1817^T
X. oryzae ICMP3125^T	100	86.31	82.62	86.61	89.23	85.57	86.15	91.14	79.58	77.92	89.96	84.89	86.54	89.89	86.44	91.21	86.88	80.03	79.42	89.61	88.77	89.78	86.43	90.00	79.61	85.96	85.58
X. codiaei CFBP4690^T	86.07	100	83.22	91.55	86.53	89.15	87.13	86.21	80.72	78.41	86.24	85.53	86.23	86.53	91.38	85.97	86.96	80.71	80.28	86.24	86.95	86.29	87.35	86.42	80.53	87.19	85.69
X. maliensis LMG27592^T	82.56	83.07	100	82.88	82.44	82.54	82.46	82.21	79.94	77.75	82.63	82.77	82.56	82.94	82.85	82.54	83.25	79.90	79.71	82.75	82.82	82.51	82.67	83.00	79.69	82.39	82.11
X. cassavae CFBP4642^T	86.40	91.63	82.97	100	86.75	89.19	87.37	86.45	80.43	78.11	86.77	85.67	86.59	87.13	93.13	86.25	87.72	80.43	80.17	86.48	87.16	86.83	87.75	87.05	80.20	87.41	85.90
X. bromi LMG947^T	89.29	86.58	82.58	86.82	100	86.04	86.85	89.47	79.95	77.89	89.22	85.15	86.65	89.16	86.89	89.41	87.00	80.10	79.63	89.17	88.77	89.21	86.89	89.13	79.79	86.91	85.97
X. cucurbitae CFBP2542^T	85.45	89.18	82.75	89.09	86.08	100	86.47	85.47	80.14	77.91	85.71	85.10	85.60	85.85	89.49	85.50	86.25	80.28	79.97	85.55	86.19	85.68	86.93	85.90	79.93	86.65	85.03
X. dyei CFBP7245^T	86.08	87.03	82.48	87.27	86.77	86.42	100	86.47	79.93	77.87	86.19	85.50	86.75	86.36	88.02	86.02	86.93	80.16	79.60	85.97	87.31	86.23	91.06	86.24	79.77	90.32	86.20
X. prunicola CFBP8353^T	91.03	86.13	82.34	86.38	89.28	85.37	86.53	100	79.56	77.42	89.57	85.01	86.61	89.65	86.62	90.85	87.02	79.70	79.15	89.30	88.65	89.60	87.56	89.61	79.34	86.33	85.96
X. hyacinthi CFBP1156^T	79.61	80.67	79.97	80.29	79.87	80.22	79.90	79.63	100	83.63	79.88	80.09	80.00	80.01	80.36	79.40	80.64	92.31	87.59	79.77	80.05	79.94	80.12	79.98	90.47	79.78	79.62
X. albilineans CFBP2523^T	77.96	78.46	78.02	78.26	77.85	77.95	77.97	77.55	83.78	100	77.99	78.09	78.03	78.04	78.21	77.83	78.36	83.47	84.15	77.95	78.05	77.97	78.08	78.02	83.31	78.01	77.68
X. citri LMG9322^T	89.77	86.27	82.74	86.79	89.14	85.72	86.25	89.63	79.94	77.89	100	85.03	86.49	93.90	86.43	89.82	87.02	79.89	79.63	92.80	88.70	98.26	86.54	93.95	79.74	86.04	85.97
X. campestris ATCC33913^T	84.93	85.48	82.78	85.61	85.11	85.02	85.47	85.05	80.08	77.95	85.05	100	85.87	85.16	85.61	84.90	86.17	80.39	79.84	84.89	85.54	85.17	85.46	85.15	79.89	85.28	85.13
X. cynarae CFBP4188^T	86.38	86.24	82.64	86.54	86.56	85.58	86.84	86.58	79.97	77.93	86.50	85.81	100	86.54	86.45	86.32	89.39	80.17	79.58	86.28	87.40	86.56	86.99	86.42	79.75	86.27	90.68
X. euvesicatoria LMG27970^T	89.96	86.61	83.17	86.94	89.17	85.86	86.41	89.67	80.19	78.18	93.86	85.28	86.67	100	86.65	89.92	87.12	80.42	79.98	92.74	88.69	93.98	86.67	98.53	80.12	86.21	86.09
X. floridensis WHRI8848^T	86.24	91.48	82.99	93.10	86.84	89.53	88.01	86.57	80.38	78.17	86.47	85.60	86.46	86.67	100	86.11	87.17	80.52	80.05	86.18	87.21	86.44	88.88	86.55	80.08	87.89	85.82
X. vasicola CFBP2543^T	91.16	86.06	82.56	86.33	89.36	85.39	86.05	90.98	79.47	77.74	89.94	84.92	86.35	89.96	86.21	100	86.69	79.81	79.25	89.42	88.69	89.91	86.29	90.01	79.28	85.98	85.84
X. arboricola CFBP2528^T	86.71	86.92	83.24	87.65	86.92	86.29	86.98	87.06	80.64	78.26	86.91	86.12	89.41	86.96	87.20	86.65	100	80.66	80.23	86.63	87.59	87.00	87.79	86.88	80.31	86.77	88.49
X. translucens DSM18974^T	79.83	80.61	80.05	80.34	79.91	80.19	80.06	79.88	92.23	83.50	79.88	80.21	80.01	80.26	80.40	79.75	80.67	100	86.99	79.91	80.23	80.15	80.20	79.94	89.83	79.95	79.74
X. sacchari CFBP4641^T	79.42	80.30	79.87	80.12	79.46	79.89	79.60	79.37	87.43	84.17	79.73	79.83	79.57	79.95	80.01	79.22	80.28	87.02	100	79.52	79.71	79.72	79.87	79.76	86.70	79.36	79.16
X. axonopodis DSM3585^T	89.45	86.23	82.86	86.47	89.12	85.49	86.10	89.33	79.68	77.94	92.80	84.93	86.28	92.75	86.27	89.40	86.67	79.98	79.53	100	88.49	92.69	86.30	92.84	79.74	85.95	85.67
X. nasturtii WHRI8853^T	88.73	87.01	82.81	87.11	88.86	86.32	87.42	88.74	80.07	77.96	88.74	85.65	87.41	88.69	87.21	88.72	87.59	80.35	79.74	88.58	100	88.71	87.66	88.70	79.87	87.39	86.68
Pseudomonas cissicola CCUG 18839^T	89.80	86.23	82.62	86.73	89.13	85.70	86.22	89.65	79.90	77.84	98.30	85.07	86.60	94.04	86.47	89.97	86.97	80.34	79.67	92.66	88.62	100	86.49	93.98	79.88	86.01	85.96
X. pisi DSM18956^T	86.31	87.37	82.82	87.66	86.88	86.85	91.14	87.61	80.06	77.89	86.53	85.52	86.99	86.61	88.99	86.23	87.76	80.22	79.66	86.45	87.63	86.56	100	86.60	79.98	90.10	86.31

X. perforans DSM18975[T]	89.87	86.51	83.09	87.02	89.10	85.87	86.32	89.73	79.98	78.00	94.00	85.16	86.54	98.55	86.57	89.93	86.91	80.12	79.72	92.90	88.77	94.03	86.58	100	79.94	86.17	86.00
X. theicola CFBP4691[T]	79.79	80.62	79.86	80.39	79.79	80.02	79.91	79.55	90.41	83.32	79.81	79.97	79.68	80.10	80.51	79.54	80.45	89.99	86.97	80.00	80.22	79.94	80.31	80.08	100	79.84	79.55
X. vesicatoria LMG911[T]	85.89	87.23	82.46	87.41	86.79	86.53	90.35	86.30	79.79	77.77	86.06	85.24	86.29	86.08	87.90	85.86	86.78	79.98	79.40	85.92	87.34	86.08	90.12	86.07	79.56	100	85.69
X. populi CFBP1817[T]	85.79	85.66	82.23	85.94	85.91	84.96	86.13	85.97	79.63	77.68	85.92	85.13	90.77	86.11	85.82	85.82	88.51	79.77	79.22	85.74	86.68	86.06	86.26	85.97	79.63	85.68	100
