# Supplementary figures and images for: Phylogenomic Analyses of the Genus Pseudomonas Lead to the Rearrangement of Several Species and the Definition of New Genera
Source: Biology (Basel). 2021 Aug 16;10(8):782. doi: 10.3390/biology10080782 (PMC8389581; doi:10.3390/biology10080782)

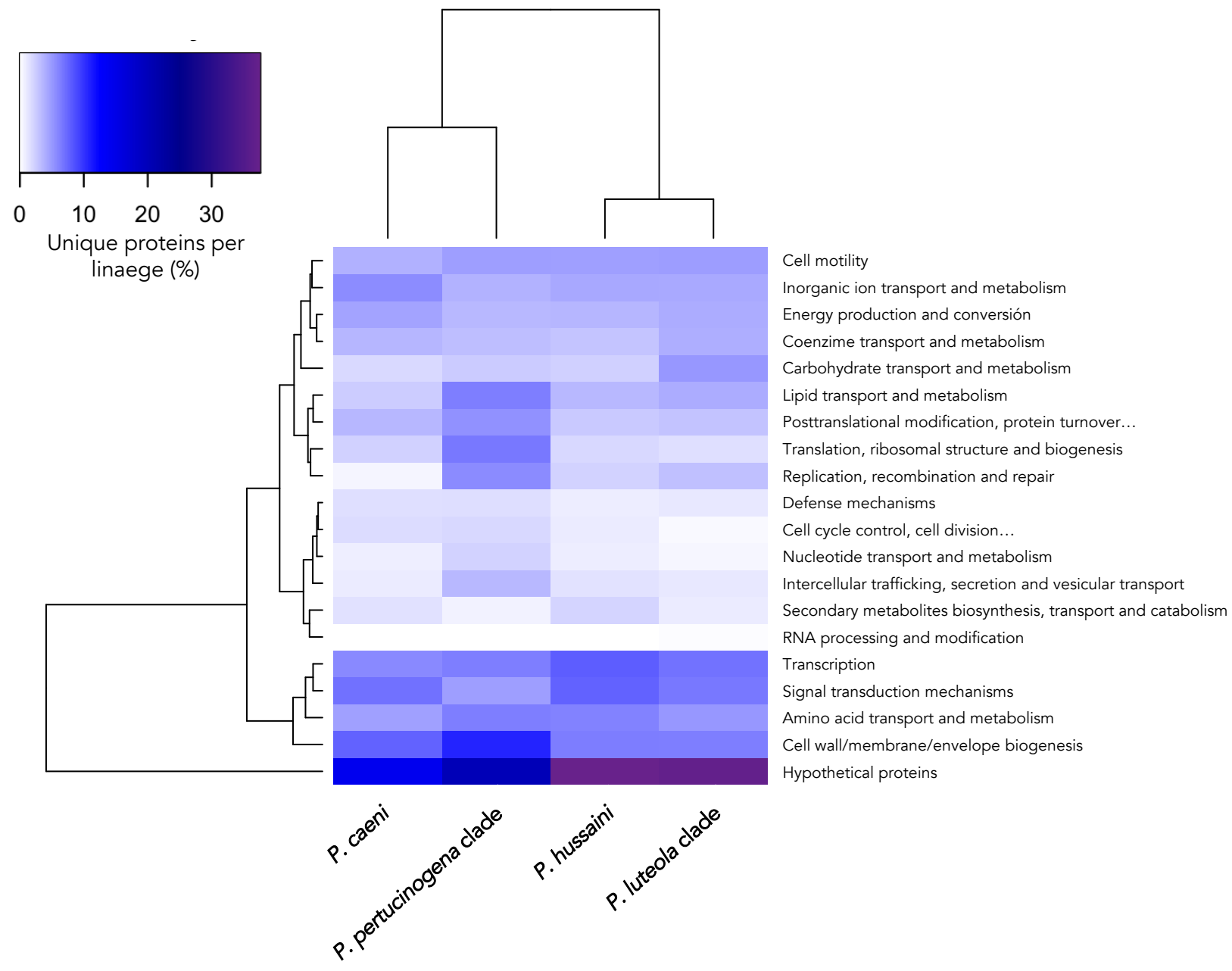

Supplement: Supplementary file 1 [file biology-10-00782-s001.zip › biology-1314851/supplementary files/Figure S1.pdf]
